# Differentiated extracts from freshwater and terrestrial mollusks inhibit virulence factor production in *Cryptococcus neoformans*

**DOI:** 10.1038/s41598-023-32140-3

**Published:** 2023-03-26

**Authors:** Davier Gutierrez-Gongora, Fouad Raouf-Alkadhimi, Ryan S. Prosser, Jennifer Geddes-McAlister

**Affiliations:** 1 Molecular and Cellular Biology Department, University of Guelph, Guelph, ON Canada; 2 Department of Environmental Toxicology, University of Guelph, Guelph, ON Canada

**Keywords:** Antifungal agents, Pathogens, Proteomics

## Abstract

The human fungal pathogen, *Cryptococcus neoformans*, is responsible for deadly infections among immunocompromised individuals with the evolution of antifungal resistance driving the solution to discover new compounds that inhibit fungal virulence factors rather than kill the pathogen. Recently, exploration into natural sources (e.g., plants, invertebrates, microbes) of antifungal agents has garnered attention by integrating a One Health approach for new compound discovery. Here, we explore extracts from three mollusk species (freshwater and terrestrial) and evaluate effects against the growth and virulence factor production (i.e., thermotolerance, melanin, capsule, and biofilm) in *C. neoformans*. We demonstrate that clarified extracts of *Planorbella pilsbryi* have a fungicidal effect on cryptococcal cells comparable to fluconazole. Similarly, all extracts of *Cipangopaludina chinensis* affect cryptococcal thermotolerance and impair biofilm and capsule production, with clarified extracts of *Cepaea nemoralis* also conveying the latter effect. Next, inhibitory activity of extracts against peptidases related to specific virulence factors, combined with stress assays and quantitative proteomics, defined distinct proteome signatures and proposed proteins driving the observed anti-virulence properties. Overall, this work highlights the potential of compounds derived from natural sources to inhibit virulence factor production in a clinically important fungal pathogen.

## Introduction

Fungal diseases continue to be an underestimated problem despite extensive impact on global health^1^. For instance, during the last decade, the human fungal pathogen, *Cryptococcus neoformans*, has been responsible for 19% of deaths worldwide among individuals with HIV/AIDS^2^. To treat cryptococcal infections, different classes of antifungal agents are available, including polyenes (e.g., amphotericin B), flucytosine (5FC), and azoles (e.g., fluconazole)^3^; however, prolonged treatment regimens (e.g., 6–12 months for azoles) in the clinic and preventative use in the field contribute to an increased prevalence of resistant strains^4,5^. This issue stresses the urgent need for development of novel treatment options less prone to the evolution of resistance to combat *C. neoformans* and other fungal pathogens. However, this can be a daunting task given the close evolutionary relationships between the fungi and host with shared phenotypic characteristics (e.g., organelles) and biochemical pathways^3^.

In this context, *C. neoformans* produces and secretes a variety of virulence factors that enable the fungus to infect and spread throughout the human body^6^. Typically, these factors are not related to fungal growth, which highlights the potential use as antifungal targets that are less prone to developing resistance by imposing a lower selective pressure^7,8^. For example, the production of melanin (protection against the host’s temperature and oxidative stress), biofilms (protection against antifungal compounds and the host immune response), polysaccharide capsule (impairment of the host immune response), and extracellular enzymes (degradation of tissue for nutrient acquisition) influence fungal adaptability and survivability^3^. To regulate production of these virulence factors and modulate antifungal resistance, *C. neoformans* produces at least seven unique peptidases (i.e., intra- or extra-cellular enzymes that degrade proteins or peptides)^9^. These peptidases represent desirable targets for inhibition to suppress fungal virulence and prevent or treat infection.

Previous studies with clinically approved compounds indicate that peptidase inhibitors, such as HIV-1 protease inhibitors (aspartic inhibitors) or bortezomib (proteasome inhibitor used in the treatment of myeloma), have potential anti-cryptococcal activity^10,11^. However, these and other synthetically-derived inhibitors can be unstable and have toxic effects on the host^12,13^. Conversely, naturally sourced compounds (e.g., plants, invertebrates, microbes) can avoid these undesirable characteristics based on years of evolution against multiple pathogens without prohibitory effects towards the host^9,14,15^. For instance, invertebrates (e.g., mollusks) rely on an innate immune system to prevent infections from the environment, demonstrating antimicrobial and antiviral effects of invertebrate-derived peptidase inhibitors^16–19^. As a result, using a One Health approach, which integrates environmental, animal, and human health for the discovery of naturally sourced compounds represents an innovative strategy for overcoming fungal infection and disrupting the cycle of antifungal resistance^20,21^.

In this study, we aim to assess the potency of complex extracts from natural sources against *C. neoformans* and propose new opportunities to impair virulence of the pathogen. We extracted proteins and other unidentified molecules in crude and clarified forms from two species of freshwater (*Planorbella pilsbryi* and *Cipangopaludina chinensis*) and one species of terrestrial (*Cepaea nemoralis*) mollusks and assessed impact of the extracts on virulence factor production by *C. neoformans*. Notably, *P. pilsbryi* and *C. nemoralis* are pulmonated snail species that are native to Canada whereas, *C. chinensis* is a prosobranch snail that is native to East Asia but has become established across North America^22^. We observed considerable effects on fungal growth, thermotolerance, biofilm formation and disruption, as well as capsule production dependent upon source, clarification process, and extract concentration, but no effect on melanin was detected nor a substantiated cytotoxic effect towards immortalized macrophages. Additionally, we performed inhibitory activity assays against multiple peptidases related to these virulence factors followed by stress and secretion assays to narrow down the inhibitory mechanisms for each extract. We combine these phenotypic findings with observed protein signatures from each extract putatively associated with the observed antimicrobial properties via mass spectrometry-based proteomics profiling^23,24^. Integration of phenotypic characterization with functional protein classification highlights common and unique responses across the mollusk extracts and proposes candidate proteins produced by invertebrates in the natural environment with antimicrobial roles against *C. neoformans*. Although, pure active molecules are not described within these findings, the potential for harvesting natural sources with beneficial antimicrobial properties is highly promising.

## Material and methods

### Mollusk extraction

Extracts from two species of freshwater snails, *P. pilsbryi* (file ramshorn snail) and *C. chinensis* (Chinese mystery snail) and, one species of terrestrial snail, *C. nemoralis* (brown-lipped snail) were prepared. *P. pilsbryi* were obtained from a continuous culture that is maintained in the School of Environmental Sciences at the University of Guelph^25^. *C. chinensis* were collected from a site on the Speed River downstream of the City of Guelph. *C. nemoralis* were collected from along Howitt Creek in Guelph. Notably, as established by the Canadian Council on Animal Care, collection and/or research of these species do not follow animal use protocols but collections were performed with minimal disruption to natural habitats^26^. Mollusk extraction was performed as previously described by our lab^27^. Briefly, tissue from mollusks was separated from the shells, 12 – 15 g of tissue was pooled, cut into small pieces, flash frozen using liquid nitrogen, and ground with a pestle and mortar. The resulting powder was resuspended in milliQ water using a 1:2 ratio (*w*:*v*) with metal beads and further disrupted using a bullet blender at 1200 rpm for 5 min at 4 °C. Next, samples were centrifuged at 12,000×*g* for 20 min at 4 °C obtaining a crude extract (i.e., non-clarified). For preparation of clarified extracts, crude extracts were subjected to thermal precipitation at 60 °C for 30 min, cooled on ice for 20 min, and centrifuged at 15,000×*g* for 45 min at 4 °C. All samples were filtered with 0.22 μm membranes, aliquoted, and stored at − 20 °C until use. Concentrations for each extract was measured by Bicinchoninic Acid Assay (BCA) (Sigma)^28^.

### Strains and growth conditions

*Cryptococcus neoformans* variety *grubii* strain H99 (Wild type) was used for all cultures. Fungal strains were routinely maintained on yeast peptone dextrose (YPD) agar (1% yeast extract, 2% Bacto-peptone, 2% D-glucose, 2% agar) and stored at 4 °C. Overnight cultures were inoculated with a single colony from the YPD plate and grown in liquid YPD medium (~ 5 mL) in a shaking incubator at 30 °C and 200 rpm.

Overnight cultures of *C. neoformans* were washed and resuspended in yeast nitrogen base (YNB) to a concentration of 10^5^ cells/mL. Next, to assess the effect of mollusk extracts (crude and clarified) on the growth of *C. neoformans* at 37 °C, 10 μL of an extract serial dilution series were mixed with 190 μL of fungal cells (previously resuspended in YNB) in a 96-well plate. Growth curves were measured using a plate reader (Synergy-H1, Biotek) at 200 rpm for 60 h at an optical density (OD) of 600 nm every 15 min. Additionally, negative controls of extract- and media-only wells were included to ensure no contamination and *C. neoformans* only wells were included to ensure proper fungal growth. All experiments were performed in five biological and two technical replicates.

### Biofilm formation

Biofilm formation was induced and measured using a previously described protocol with slight modifications^29^. Briefly, overnight *C. neoformans* cultures in YNB were resuspended in Dulbecco's Modified Eagle Medium (DMEM) (Corning) supplemented with 4.5 g/L of glucose and glutamine to a concentration of 10^7^ cells/mL. Next, 285 μL of fungal cells were combined with 15 μL of protein extracts from a serial dilution and transferred into individual wells of sterile, polystyrene, flat-bottom, 24-well microtiter plates (Corning). Wells with DMEM- or fungal cells-only were included as controls. Plates were statically incubated at 37 °C for 48 h and covered with aluminum foil (to avoid media evaporation). After the incubation period, the supernatant from each well was removed and the well was washed twice with sterile water and air-dried for 10 min at room temperature.

For biofilm quantification, 100 μL of 0.2% crystal violet solution was added to each well (including the media-only control wells) and plates were incubated at room temperature for 10 min. Next, each well was thoroughly washed three times with sterile water, and biofilms were destained with 200 μL of 100% ethanol for 10 min at room temperature. Finally, 75 μL of destained solution from each well was transferred to a new 96-well microtiter plate and OD_550nm_ was measured using a plate reader (Synergy-H1, Biotek). Media-only control values were subtracted from all measurements. All measurements were performed using three biological replicates and the experiment was repeated in duplicate.

### Biofilm disruption

For biofilm disruption, wells with fungal cells only were prepared as described above with static incubation in DMEM at 37 °C wrapped in aluminum foil for 24 h. At this time, 15 μL of mollusk extract (crude and clarified) was added to each well to achieve a final volume of 300 μL. The addition of PBS (instead of extracts) to biofilm wells was used as a control. Cells were statically incubated for an additional 24 h period at 37 °C wrapped in aluminum foil, followed by a crystal violet assay and OD_550nm_ measurement. All measurements were performed using three biological replicates and the experiment was repeated in duplicate.

### Capsule production

Overnight cultures of *C. neoformans* from YPD were transferred to 5 mL of YNB reaching a concentration of 10^5^ cells/mL and incubated overnight at 30 °C with 200 rpm shaking. Next, 5 mL of culture was collected by centrifugation and cells were washed twice with low iron media (LIM; 0.5% L-asparagine, 0.4% HEPES, 0.04% K_2_HPO_4_, 0.008% MgSO_4_·7H_2_O, 0.2% NaHCO_3_, and 0.025% CaCl_2_·2H_2_O) before resuspension of 10^5^ cells/mL in 5 mL LIM or LIM containing 100 μL of mollusk extracts (crude and clarified). Cultures were incubated for 72 h at 37 °C and 200 rpm followed by centrifugation at 800 ×*g* for 1 min with gentle washing and resuspension in 1 mL of PBS (pH 7.3). To visualize the capsule, cells were mixed with India ink using a 1:1 ratio on microscope slides and observed using a Differential Interference Contrast (DIC) microscope and a 63X oil objective. Capsule production was quantified using a ratio of total cell size (with capsule) to cell size (without capsule) All measurements were obtained using three biological replicates with 50–90 cells assessed per condition and the experiment was performed in technical duplicate.

### Glucuronoxylomannan (GXM) shedding

GXM secretion in the media was assessed as previously described with minor changes^30^. Briefly, capsule production of *C. neoformans* H99 was performed as described in section "Capsule production". After 72 h incubation in LIM, culture supernatant was centrifuged at 15,000 ×*g* for 3 min. Supernatant was collected and diluted to OD_600nm_ of 1 and incubated at 70 °C for 15 min. Then, samples were subjected to electrophoresis on an agarose gel at 25 V for 16 h and transferred onto a positive charged nylon membrane (GE Healthcare). The membrane was incubated with 25 µg/L of anti-GXM (18B7) (Fischer-Scientific, USA) monoclonal antibody (diluted in 5% skim milk) shaking at 4 °C, followed by three rinses with PBS. The membrane was then incubated for 2 h in 400 µg/L of goat-anti-mouse HRP secondary antibody (Fischer-Scientific, USA), washed for 2.5 h in PBS + 0.1% Tween-20, changing the washing buffer every 20 min. Finally, bound polysaccharide was visualized by chemiluminescence on X-ray film (GE Healthcare) and quantification of band density was performed using ImageJ version 1.53 k (WT set to 1.0; *pka1*Δ acapsular strain used as a control)^31^.

### Melanin production

To induce melanin production, overnight *C. neoformans* cultures in YPD were transferred to 5 mL of YNB and incubated overnight at 30 °C and 200 rpm. Afterwards, cells were collected by centrifugation at 800 ×*g* for 1 min and washed twice with sterile PBS (pH  7.4) and then resuspended in 1 mL of PBS to reach a final concentration of 10^6^ cells/mL. Before adding the cells, 20 μL of mollusk extracts (crude and clarified) from a serial dilution series were spread on each L-3,4-dihydroxyphenyl-alanine (L-DOPA) agar plate (1.4% agar, 13 mM glycine, 30 mM KH_2_PO_4_, 10 mM MgSO_4_·7H_2_O, 5 mM glucose, 2.8 μM thiamine, 1 mM L-DOPA (Sigma)) using a swab and allowed to dry for 15 min at room temperature. Each plate was divided into six sections, with three aliquots of 5 μL of *C. neoformans* spotted onto each section. PBS was spread (pH 7.3) and used as a control. Plates were statically incubated for 72 h at 30 °C and 37 °C, with pictures taken every 24 h under standardized conditions in a photographic chamber. To quantify melanin production, photographs were analyzed as previously described^32^. Briefly, images were converted to grayscale (8-bit format) and mean gray values of cell dots were determined using ImageJ software (National Institutes of Health, USA). Values were normalized using the background. All measurements were obtained using three biological and two technical replicates.

### Inhibition of proteolytic activity

#### Substrates, enzymes, and buffers

For enzymatic assays, Carboxypeptidase D (EC 3.4.16.6), Kexin (EC 3.4.21.61), Pepsin (EC 3.4.23.1), Subtilisin A (EC 3.4.21.62), Papain (EC 3.4.22.2), Thermolysin (EC 3.4.24.27) and 20S Proteosome from Rat (EC 3.4.25.1) were purchased and used as targets for potential inhibitory effect of the extracts. Substrates and enzymatic activity conditions for each enzyme are summarized in Supplementary Table S1.

#### Enzymatic activity conditions

To assess the inhibitory activity of extracts, each extract was pre-incubated with the enzyme for 10 min in the corresponding buffer at room temperature (See Supplementary Table S1). To ensure unbiased parameters (e.g., IC_50_), enzymatic activity was measured by adding the corresponding substrate to a final concentration of 1 *K*_*M*_ (Michaelis–Menten constant) and monitoring the appearance of the product over time. Wavelengths of excitation and/or emission used are listed in Supplementary Table S1. Inhibitory activity was quantified using Eq. (1):

1 $$a=\frac{{v}_{i}}{{v}_{c}}$$

*a*: residual activity, ${v}_{i}$: speed (product over time) reaction in presence of extracts, ${v}_{c}$: speed (product over time) reaction without the presence of extracts (control).

An inhibitory concentration (IC_50_) value was obtained by doing a non-linear fitting to a dose-response curve with Eq. (2):

2 $$a=\frac{1}{1+\frac{[I]}{{IC}_{50}}}$$

### Stress assays

To analyze potential inhibitory mechanisms of these extracts, we assessed the capability of *C. neoformans* H99 to respond to different stressors in the presence of each extract. Oxidative, osmotic and cell-wall stress was assessed using YPD agar plates supplemented with NaCl (1.5 M), H_2_O_2_ (3 mM) and SDS (0.01%), respectively. In each case, *C. neoformans* H99 cells were grown to mid-log phase in YPD at 30 °C and 200 rpm, normalized to 10^6^ cells/mL and incubated with each extract for 4 h at 30 °C and 200 rpm. Cells were serially diluted (ten-fold) from 10^6^ to 10^1^ cells/mL and 5 µL was spotted on each plate. YPD plates supplemented with the different stressors were statically incubated at 30 °C and 37 °C. Growth was followed by taking images every 24 h for 72 h. Each experiment was performed using three biological and two technical replicates.

### Urease activity assay

To assess the effect of the extracts on secretory pathways, we evaluated the activity of urease as previously described with minor variations^33^. Briefly, *C. neoformans* H99 cells were grown to mid-log phase in YPD at 30 °C and 200 rpm, normalized to 10^6^ cells/mL and incubated with each extract for 4 h at 30 °C and 200 rpm. Cells were serially diluted (ten-fold) from 10^6^ to 10^1^ cells/mL and 5 µL was spotted on Christensen’s Urea Agar (Peptone 0.1%, Glucose 0.1%, NaCl 0.5%, KH_2_PO_4_ 0.2%, Phenol Red 0.0012%, Urea 2% and Agar 1.5%)^34^. Urea agar plates were statically incubated at 30 °C and 37 °C. Urease activity, characterized by a halo of pink color, was determined by taking images at 24 h. Each experiment was performed using three biological and two technical replicates.

### Cytotoxicity assay

To analyze the cytotoxic effect of extracts against mammalian cells, we assessed the Lactate Dehydrogenase (LDH) activity of immortalized BALB/c macrophages (generously provided by Dr. Felix Meissner, Max Planck Institute of Biochemistry) as previously described with some variations^35^. Briefly, BALB/c macrophages were normalized to 5 × 10^5^ cells/mL using DMEM supplemented with penicillin and streptomycin and statically incubated at 37 °C, 5% CO_2_ for 48 h. Macrophages were gently washed with PBS and diluted in DMEM without antibiotics. Ten microliters of each extract was combined with 1 mL DMEM containing macrophages using 24 wells plates and, statically incubated at 37 °C, 5% CO_2_ for 4 h. For total death, triton 1.2% was added to the non-treated wells and statically incubated at room temperature for 30 min. LDH substrate (NAD^+^) (Sigma-Aldrich, US) and samples was mixed using a 1:1 ratio (v:v) in 96 well plates (total volume = 100 µL) and statically incubated at room temperature for 20 min before adding 50 µL of stopping solution (Sigma-Aldrich, US). LDH activity was quantified at OD_450nm_. Media only and triton 1.2% were used as controls for extract containing wells and total death replicates. Each experiment was performed using six biological replicates.

### Mass spectrometry-based proteomics

#### Preparation of samples

Profiling of the mollusk extracts was performed as previously described for secretome samples^36^. Briefly, 100 µg of crude and clarified mollusk extracts were enzymatically digested using a trypsin/Lys-C mixture, followed by desalting and purification using STop And Go Extraction (STAGE)-tips^37^.

#### Measurement of samples

Samples were run over a 60-min chromatographic gradient with an Orbitrap Exploris 240 hybrid quadrupole-orbitrap mass spectrometer (Thermo Fisher Scientific) coupled to an Easy-nLC 1200 high-performance liquid chromatography device (Thermo Fisher Scientific). Samples were loaded onto an in-line 75-mm by 50-cm PepMap RSLC EASY-Spray column filled with 2-mm C18 reverse-phase silica beads (Thermo Fisher Scientific). Separated peptides were electro-sprayed into the mass spectrometer with a linear gradient of 3% to 20% buffer B (80% acetonitrile, 0.5% acetic acid) over a 3 h gradient, followed by a wash with 100% buffer B with a 250-nL/min flow rate. The mass spectrometer switched between one full scan and MS/MS scans of abundant peaks. Full scans (*m*/*z* 400 to 2,000) were acquired in the Orbitrap mass analyzer with a resolution of 120,000 at *m*/*z* 200.

#### Data processing

Analysis of mass spectrometry raw data files was performed using MaxQuant software (version 1.6.0.26)^38,39^. Given that these mollusk species do not have complete proteomes annotated, the search was performed using the incorporated Andromeda search engine against the proteins of *Architaenioglossa* group (24,377 sequences; 7 July 2022) for *C. chinensis*, Helicina group (86,562 sequences; 7 July 2022) for *C. nemoralis*, and *Planorbidae* for *P. pilsbryi* (33,350 sequences; 7 July 2022) from UniProt^40^ and NCBI^41^. The following parameters were included: trypsin enzyme specificity with a maximum of two missed cleavages, a minimum peptide length of seven amino acids, fixed modifications, including carbamidomethylation of cysteine, and variable modifications, including, methionine oxidation and N-acetylation of proteins and split by taxonomic ID. Peptide spectral matches were filtered using a target-decoy approach at a false-discovery (FDR) of 1% with a minimum of two peptides required for protein identification. Relative label-free quantification (LFQ) was enabled and the MaxLFQ algorithm used a minimum ratio count of 1^42^.

#### Bioinformatics

Statistical analysis and data visualization of the proteomics data were performed using Perseus (version 2.0.6.0)^43^. Data were prepared by filtering for reverse database matches, contaminants, and proteins only identified by site, followed by log_2_ transformation of LFQ intensities. Filtering for valid values (three of four replicates in at least one group) was performed, missing values were imputed from the normal distribution (width, 0.3; downshift, 1.8 standard deviations), and group values were averaged. Significant differences were evaluated by a Student’s *t*-test (p-value ≤ 0.05) with multiple-hypothesis testing correction using the Benjamini-Hochberg^44^ FDR cutoff at 0.05 with S_0_ = 1. Proteomics profiling was performed in quadruplicate.

### Statistical analysis

For phenotypic assays (growth, capsule, melanin, and biofilm), data were visualized and statistically analyzed using GraphPad Prism version 9.0 (GraphPad Software, Inc., USA; https://www.graphpad.com/). Statistical tests were performed by one-way analysis of variance (ANOVA) followed by a Dunnett’s multiple comparisons test (treatments against control). P values of $\le$ 0.05 were considered significant.

## Results

### Crude and clarified extracts from mollusks showed measurable protein concentrations

Extracts from ground mollusk tissue was assessed by BCA to determine protein concentrations (Table 1). Results confirm successful protein extraction from the natural sources and as expected, crude extracts had a higher concentration compared to clarified preparations. Notably, the highest protein concentration was from crude *C. nemoralis* extracts; for subsequent experiments, concentrations were normalized across extracts, as appropriate and dilution series were generated.

**Table 1:** Protein concentration determination for mollusk extracts.

Mollusks extracts		Concentration (mg/mL)
*Cepaea nemoralis*	Crude	11.63 ± 0.35
	Clarified	2.86 ± 0.19
*Planorbella pilsbryi*	Crude	2.90 ± 1.0
	Clarified	2.36 ± 0.23
*Cipangopaludina chinensis*	Crude	6.11 ± 0.05
	Clarified	4.28 ± 0.11

### Protein extracts from mollusks inhibit *C. neoformans* growth and thermotolerance

**Figure 1:**
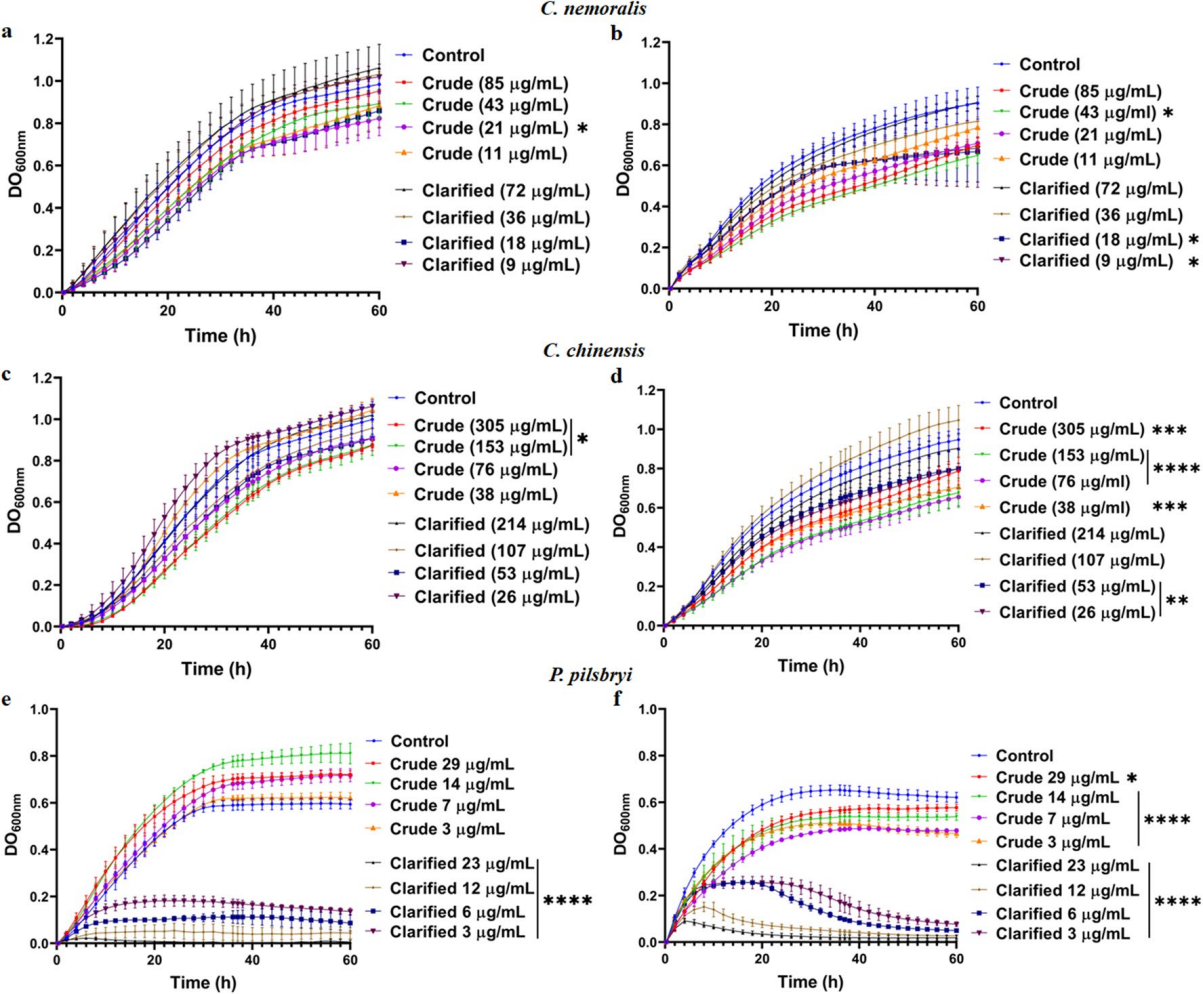
Effect of extracts from mollusks towards *C. neoformans* growth. (**a**) and (**b**) Effect of extracts from *C. nemoralis* at 30 °C and 37 °C, respectively; (**c**) and (**d**) Effect of extracts from *C. chinensis* at 30 °C and 37 °C, respectively; and (**e**) and (**f**) Effect of extracts from *P. pilsbryi* at 30 °C and 37 °C, respectively. Controls consist of *C. neoformans* cells in YNB media without extracts. Experiments performed in five biological replicates and technical duplicate. Error bars indicate standard deviation. Statistical analysis performed using a one-way ANOVA and a Dunnett’s multiple comparison test with a p-value of 0.05; *p < 0.05; **p < 0.01; ***p < 0.001 and ****p < 0.0001. Figures created using GraphPad Prism 9.

To assess potential antifungal properties of the mollusk extracts (crude and clarified) on *C. neoformans*, we exposed the fungi to a range of extract concentrations (2X dilution series from pure) at environmental (30 °C) and physiological (37 °C) temperatures. Comparison of extracts showed variable antifungal effects on the growth of *C. neoformans* in YNB media. For *C. nemoralis*, we observed minor inhibition of fungal growth in the presence of the crude extracts at 30 °C with a significant (p < 0.05) difference in growth using a low extract concentration (21 µg/mL) compared to the control (i.e., no extract present) (Fig. 1a). At 37 °C, we observed disruption to fungal thermotolerance effects with significant (p < 0.05) growth reduction for crude (43 µg/mL) and clarified (9 & 18 µg/mL) *C. nemoralis* extracts (Fig. 1b). We observed similar findings for *C. chinensis* extracts with significant (p < 0.05) inhibition at 30 °C only observed for high crude extract concentrations (305 & 152 µg/mL) (Fig. 1c). Similarly, all concentrations of crude and low concentrations of clarified (54 & 27 µg/mL) extracts showed a significant (p < 0.01; p < 0.001; p < 0.0001) reduction in thermotolerance associated with fungal growth at 37 °C (Fig. 1d). Strikingly, for clarified extracts of *P. pilsbryi* at 30 °C, we observed a consistent and significant (p < 0.0001) reduction in fungal growth across concentrations (Fig. 1e). Likewise, at 37 °C, we observed the same consistent and significant (p < 0.05; p < 0.0001) reduction in fungal growth and thermotolerance by the clarified extracts of *P. pilsbryi* with a MIC value of 23 µg/mL. Similarly, we detected a significant reduction by the crude extracts, although the impact on fungal growth was less apparent (Fig. 1f). Lastly, we assessed the toxicity of the crude and clarified extracts towards immortalized macrophages; we did not observe a significant cytotoxic effect using extract concentrations within the range of inhibition of *C. neoformans* growth except for the *C. chinensis* clarified sample (Supplementary Fig. S1). Overall, exposure of *C. neoformans* to crude and clarified mollusk extracts impacted fungal growth and thermotolerance to varying degrees; however, clarified *P. pilsbryi* extracts showed the most consistent and noticeable effect across temperatures and concentrations. Further, cytotoxicity of extracts towards macrophages was minimal, supporting the potential for therapeutic applications.

### Protein extracts from* C. chinensis *inhibited biofilm formation and disrupted pre-stablished *C. neoformans* biofilms

**Figure 2:**
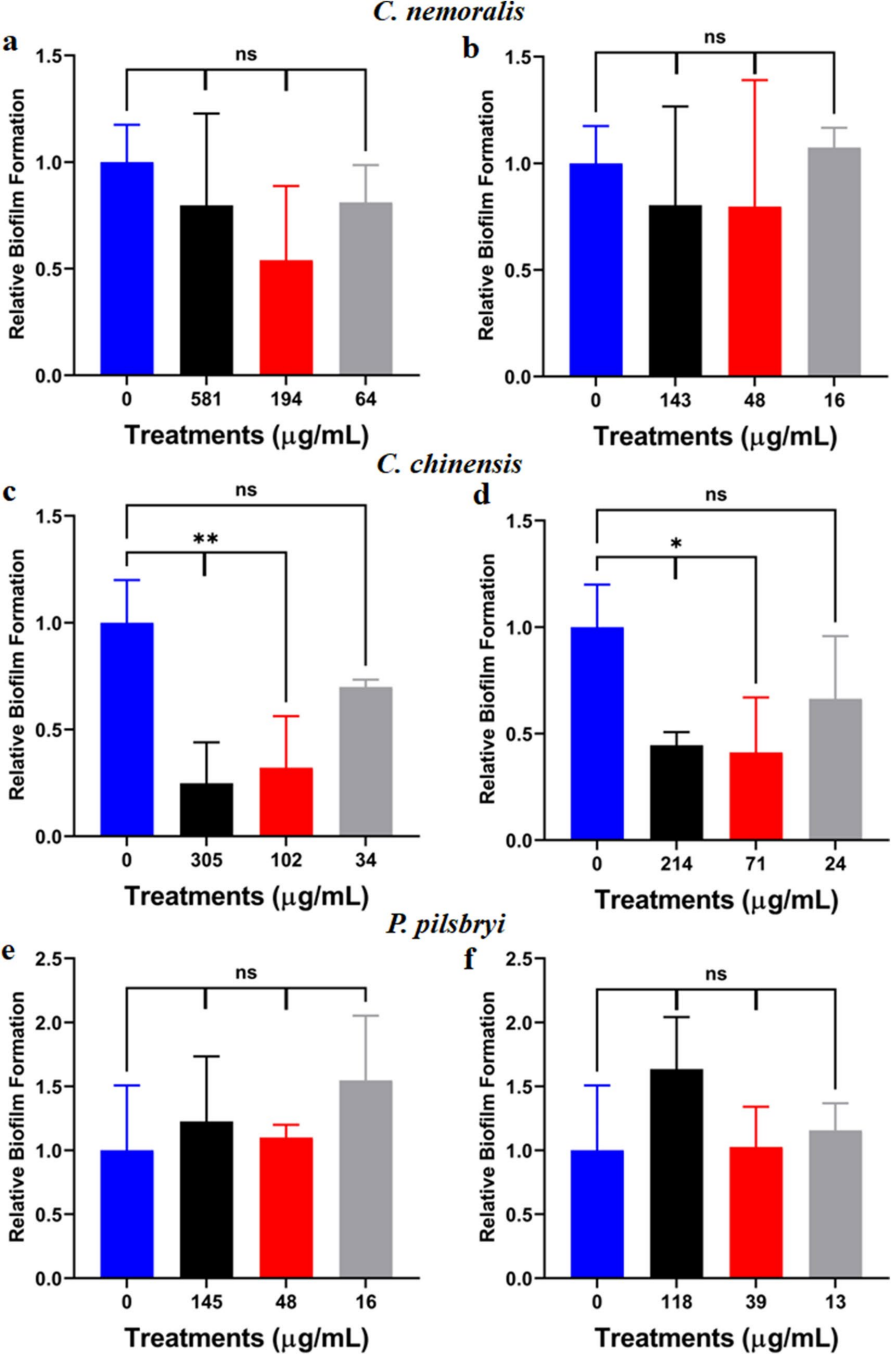
Changes to *C. neoformans* biofilm formation in the presence of mollusk extracts at 37 °C. (**a**) and (**b**) Effect of crude and clarified extracts from *C. nemoralis*, respectively; (**c**) and (**d**) Effect of crude and clarified extracts from *C. chinensis*, respectively and (**e**) and (**f**) Effect of crude and clarified extracts from *P. pilsbryi*, respectively. Biofilm formation on each condition was normalized to a control without any extract (0 µg/mL). Experiments were performed in biological triplicate and technical duplicate. Error bars indicate standard deviation. Statistical analysis was performed using a one-way ANOVA and a Dunnett’s multiple comparison test with a p-value of 0.05. **p < 0.01; ***p < 0.001 and ****p < 0.0001. Figures were created using GraphPad Prism 9.

To evaluate the putative effects of mollusk extracts on cryptococcal biofilm formation, biofilms were established over a 48-h period of growth along with incubation with crude or clarified extracts from *C. nemoralis, C. chinensis,* and *P. pilsbryi* at 37 °C. Across all concentrations of *C. nemoralis* crude (Fig. 2a) and clarified (Fig. 2b) extracts we did not observe changes in biofilm formation. Conversely, for *C. chinensis,* we defined a significant (p < 0.05; p < 0.01) dose-dependent effect against *C. neoformans* biofilm formation at the higher concentrations for both crude (Fig. 2c) and clarified (Fig. 2d) extracts. Extracts from *P. pilsbryi* mirrored the lack of effects observed for *C. nemoralis* (Figs. 2e, 2f).

**Figure 3:**
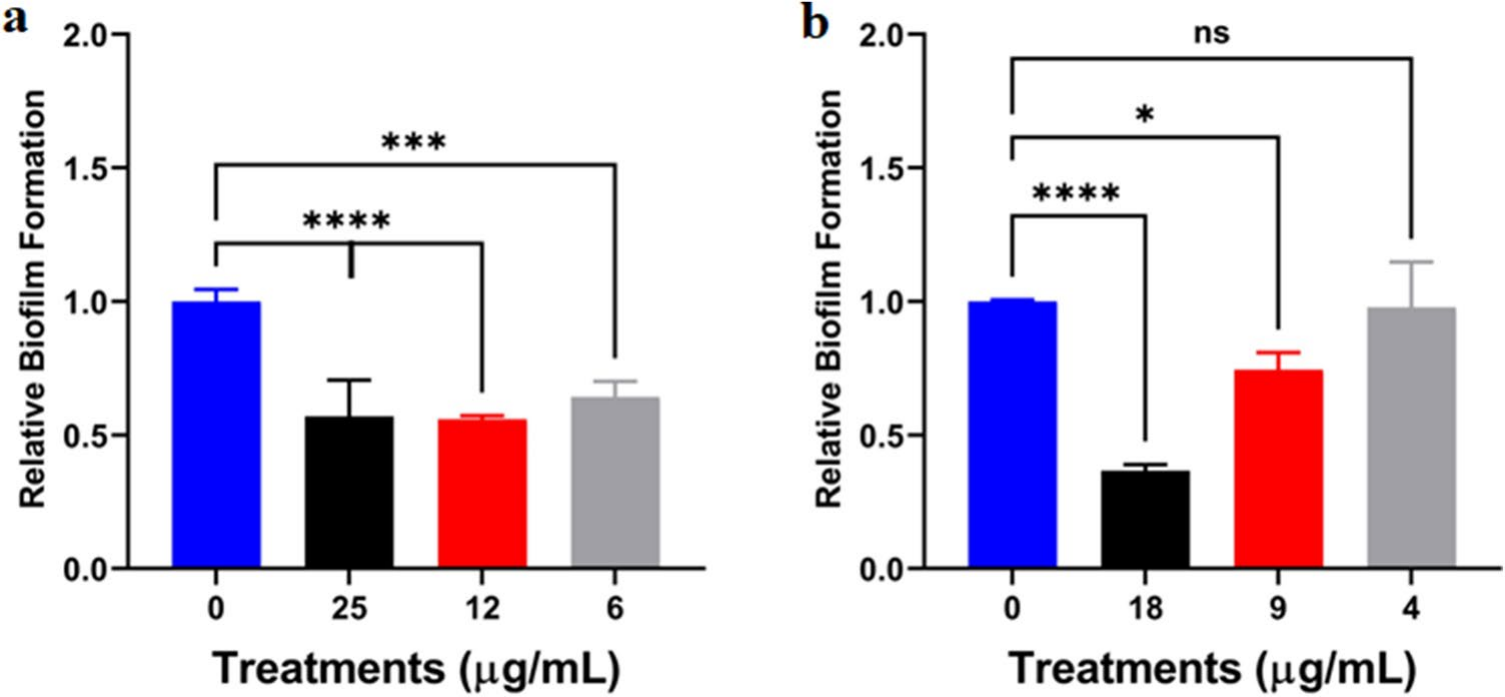
Relative effect of extracts from *C. chinensis* on pre-formed biofilms of *C. neoformans* at 37 °C. (**a**) Effect of crude extracts; and (**b**) Effect of clarified extracts. Cryptococcal cells grew on DMEM supplemented with glucose for 24 h before extracts were applied. Biofilm formation on each condition was normalized to control without any extract (0 µg/mL). Experiments were performed in biological triplicate and technical duplicate. Error bars indicate standard deviation. Statistical analysis was performed using a one-way ANOVA and a Dunnett’s multiple comparison test with a p-value of 0.05. **p < 0.01; ***p < 0.001 and ****p < 0.0001. Figures were created using GraphPad Prism 9.

Given our observation that *C. chinensis* inhibits biofilm formation, we assessed if the crude and clarified extracts were able to disrupt pre-formed fungal biofilms at 37 °C. We observed that crude extracts of *C. chinensis* significantly (p < 0.001; p < 0.0001) disrupted biofilm formation across all concentrations evaluated at physiological temperature (Fig. 3a). Similarly, the clarified extracts showed significant (p < 0.05; p < 0.0001) disruption to fungal biofilms at two of the tested extract concentrations (18 and 9 µg/mL) (Fig. 3b). Together, we determined that crude and clarified *C. chinensis* extracts inhibit and disrupt *C. neoformans* biofilms at 37 °C.

### Extracts from *C. nemoralis* and *C. chinensis* inhibit capsule production of *C. neoformans*

**Figure 4:**
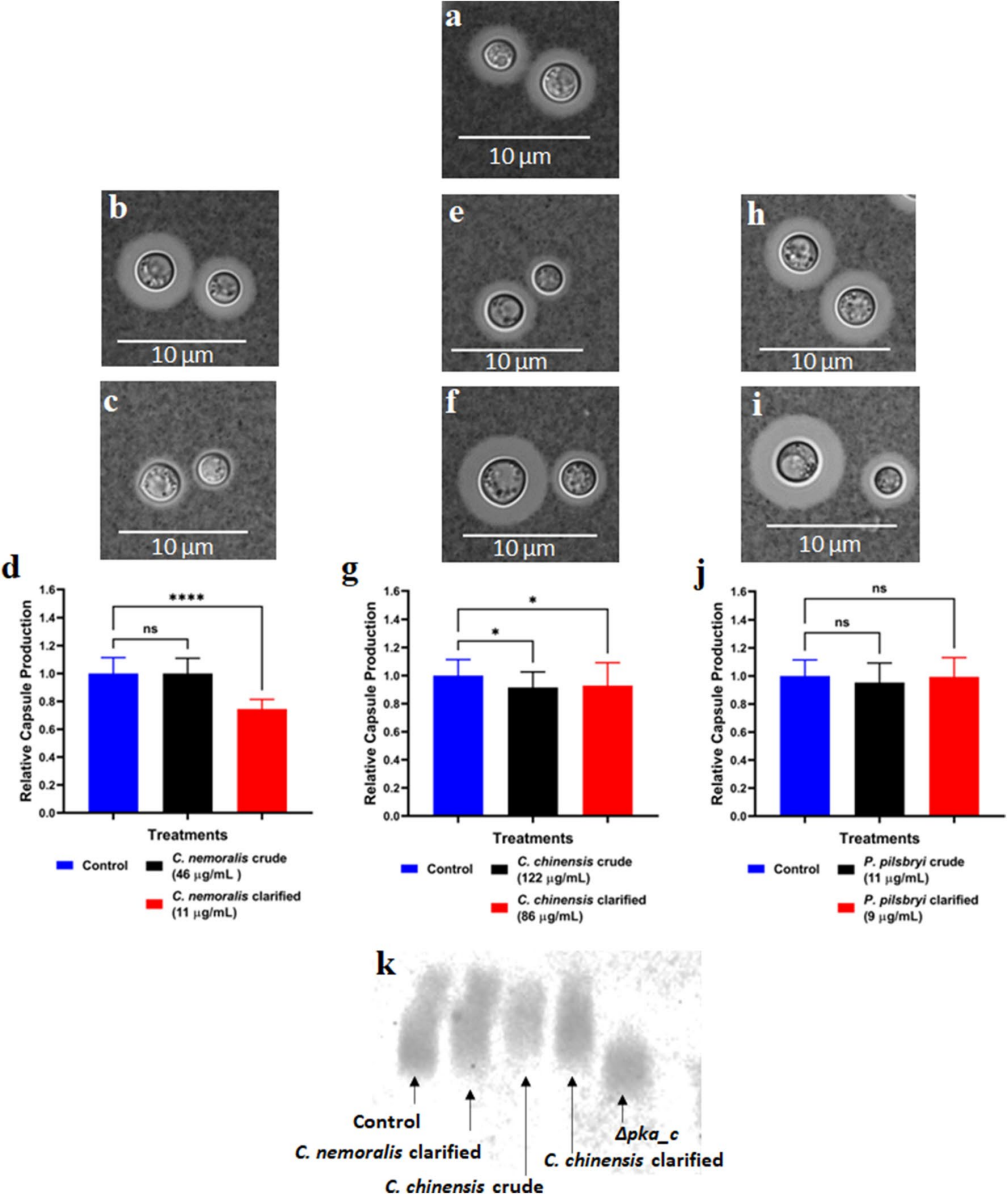
Representative capsule production of *C. neoformans* in presence of mollusk extracts at 37 °C. (**a**) Control (LIM and *C. neoformans* H99 only); (**b**) and (**c**) Effect of crude (46 µg/mL) and clarified (11 µg/mL) extracts from *C. nemoralis*, respectively; (**e**) and (**f**) Effect of crude (122 µg/mL) and clarified (86 µg/mL) extracts from *C. chinensis*, respectively and (**h**) and (**i**) Effect of crude (11 µg/mL) and clarified (9 µg/mL) extracts from *P. pilsbryi*, respectively. (**d**), (**g**) and (**j**) Statistical analysis between treatments and control. (**k**) Visualization of GXM released to the extracellular space. Control and treatments were performed using *C. neoformans* H99 cells and the acapsular *C. neoformans* strain, Δ*pka,* as a negative control. Cells were visualized using India ink (1:1 ratio) and a DIC microscope with an oil 63X objective. Error bars indicate standard deviation. Statistical analysis was performed using a one-way ANOVA and a Dunnett’s multiple comparison test with a p-value of 0.05. **p < 0.01; ***p < 0.001 and ****p < 0.0001. Approx. 50–60 cells were measured per treatment, with ratio of total cell (including capsule) to cell body presented. Experiment was performed in biological triplicate and technical duplicate. Figures were created using GraphPad Prism 9.

To assess the effects of the mollusk extracts on another critical virulence factor of *C. neoformans*, we explored inhibition of polysaccharide capsule production. We used *C. neoformans* H99 cultured in LIM as the control (Fig. 4a) for comparison to extract treatments. *C. nemoralis* extracts were included in the LIM incubation with no change in capsule production for the crude extract (Fig. 4b), whereas a significant (p < 0.0001) reduction in capsule (25%) was observed with the clarified extract (11 µg/mL) (Fig. 4c); these phenotypic observations were confirmed by quantification of the ratio of total cell size against cell body size (Fig. 4d). Similarly, for *C. chinensis*, a significant (p < 0.05) reduction in capsule formation (approx. 10%) was observed upon treatment with the crude (Fig. 4e) and clarified (Fig. 4f) extracts followed by quantification (Fig. 4g). However, for *P. pilsbryi*, there was no reduction in fungal capsule production observed in the presence of crude or clarified extracts (Fig. 4h-j). Assessment of capsule shedding showed that treatment of cryptococcal cells with *C. nemoralis* clarified did not have a substantial effect on GXM secretion compared to WT (control) (Fig. 4k). Conversely, a reduction in capsule shedding was observed upon treatment of the cryptococcal cells with *C. chinensis* crude and clarified extracts relative to WT, supporting the microscopy findings. An assessment of relative band density compared to WT (set to 1.0), quantified capsule production at 0.91 for *C. nemoralis* clarified, 0.63 for *C. chinensis* crude, 0.64 for *C. chinensis* clarified, and 0.58 for *pka1*Δ (acapsular control strain). These data define a notable effect against *C. neoformans* capsule production using different mollusk species and extraction procedures, which supports an opportunity to weaken fungal virulence upon extract treatment.

### Mollusk extract did not affect melanin production of *C. neoformans*

**Figure 5:**
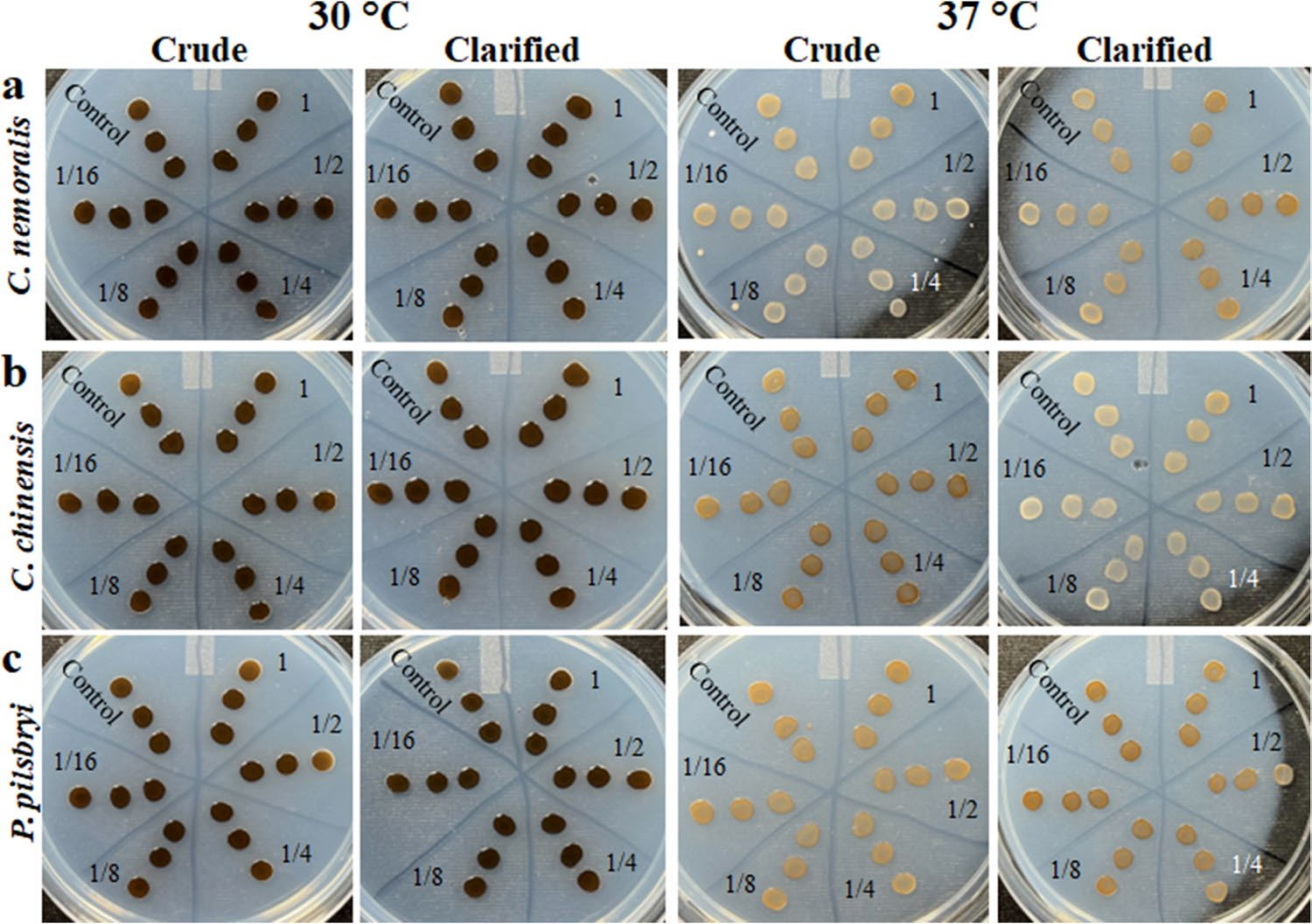
Effect of mollusk extracts on melanin production of *C. neoformans* at 30 °C and 37 °C. Row (**a**) *C. nemoralis* crude extracts (starting concentration: 2.1 mg/mL) and clarified (starting concentration: 0.6 mg/mL). Row (**b**) *C. chinensis* crude extracts (starting concentration: 1.5 mg/mL) and clarified (starting concentration: 0.4 mg/mL). Row (**c**) *P. pilsbryi* crude extracts (starting concentration: 0.5 mg/mL) and clarified (starting concentration: 0.3 mg/mL). For control, only PBS (pH 7.3) was spread on the plate. Images were taken after 48 h of incubation at each temperature. Experiment performed in biological triplicate and technical duplicate.

To explore a potential impact of mollusk extracts on the production of melanin by *C. neoformans*, L-DOPA plates were prepared. Notably, we did not observe a measurable (p > 0.05) change in melanin production at 30 °C or 37 °C in the presence of crude and clarified extracts from *C. nemoralis* (Fig. 5a), *C. chinensis* (Fig. 5b), and *P. pilsbryi* (Fig. 5c) relative to the untreated control. This experiment was repeated in triplicate and performed using multiple plate assays (i.e., dilution plates, spot assays) (data not shown) and consistently showed the same results.

### Mollusk extracts demonstrate an inhibitory effect against virulence-related peptidases

Given the role of peptidases in cryptococcal virulence^9^, we evaluated the effect of each mollusk extract (crude and clarified) by IC_50_ values for a suite of cryptococcal virulence-related peptidases (i.e., Carboxypeptidase D (EC 3.4.16.6), Kexin (EC 3.4.21.61), Pepsin (EC 3.4.23.1), Subtilisin A (EC 3.4.21.62), Papain (EC 3.4.22.2), Thermolysin (EC 3.4.24.27), 20S Proteosome from Rat (EC 3.4.25.1)) (Table 2). Notably, extracts were tested for inhibitory activity against enzymes connected to the phenotypic profiling observed in this study. Here, we defined inhibitory activity of *C. nemoralis* and *P. pilsbryi* crude extracts against Subtilisin A (associated with fungal quorum sensing), Kexin-like (associated with melanin production), and Thermolysin (associated with brain invasion). Clarified *C. nemoralis* showed inhibitory activity against Cysteine-like or Papain (associated with capsule production), whereas clarified *P. pilsbryi* inhibited Subtilisin A. For *C. chinensis*, the crude and clarified extracts showed inhibitory activity against Subtilisin A with the clarified extract inhibiting Pepsin (associated with biofilm). For assays with no measurable effect, these are indicted by ‘NE’ and if no change in fungal phenotypic profiling was observed for the respective extract and preparation, activity was not assessed (i.e., ‘ND’). Together, these findings align many inhibitory activity profiles of the extracts with the phenotypic observations associated with changes in fungal virulence factor production.

**Table 2:** IC_50_ values of mollusk extracts towards virulence-related peptidases defined in *Cryptococcus neoformans*.

Protein extracts		Subtilisin-like (Quorum sensing)	Kexin-like (Melanin)	Cysteine-like (Capsule)	Pepsin-like (Biofilm)	Thermolysin-like (Brain invasion)
*Cepaea nemoralis*	Crude	2.5 µg/mL	72.2 µg/mL	NE	ND	22 µg/mL
	Clarified	ND	NE	57.2 µg/mL	ND	ND
*Planorbella pilsbryi*	Crude	1.9 µg/mL	61.4 µg/mL	ND	ND	48 µg/mL
	Clarified	18.88 µg/mL	ND	ND	ND	ND
*Cipangopaludina chinensis*	Crude	5.3 µg/mL	ND	ND	NE	ND
	Clarified	4.43 µg/mL	ND	ND	15.55 µg/mL	ND

*ND* not determined, *NE* no effect.

### Mollusk extracts impair ability of *C. neoformans* to adapt to external stressors but do not influence secretory pathways as defined by urease production

**Figure 6:**
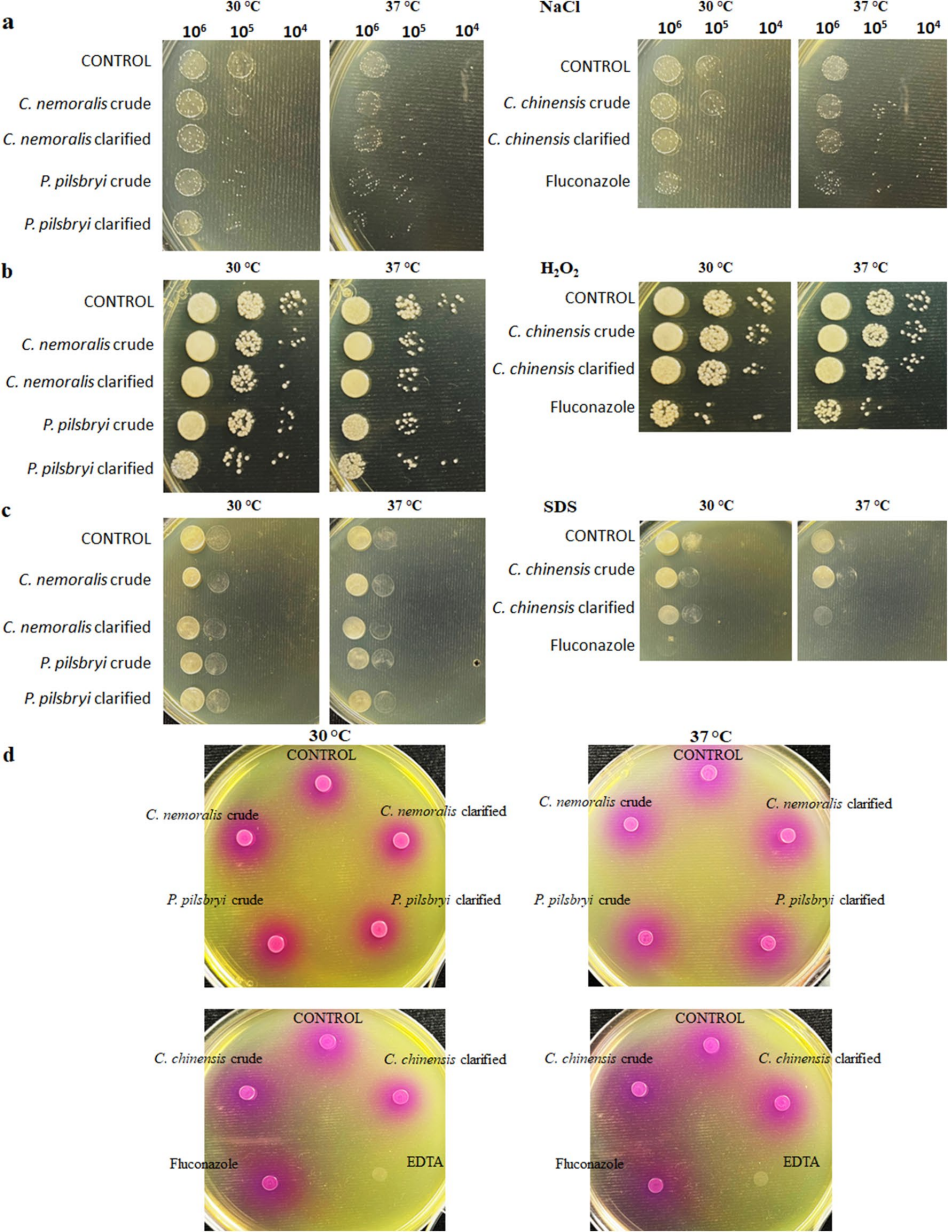
Effect of protein extracts on stress response and urease production for *C. neoformans*. Images show *C. neoformans* growth in YPD-agar supplemented with different stressors or Christensen’s Urea Agar after 4 h incubation with protein extracts from *C. nemoralis* crude (29 µg/mL), *C. nemoralis* clarified (35 µg/mL), *C. chinensis* crude (160 µg/mL), *C. chinensis* clarified (40 µg/mL), *P. pilsbryi* crude (40 µg/mL) and *P. pilsbryi* clarified (40 µg/mL). (**a**) Osmotic stress was performed with NaCl at 1.5 M. (**b**) Oxidative stress was performed with H_2_O_2_ at 3 mM. (**c**) Membrane stress was performed with SDS 0.01%. (**d**) Urease secretion assay. Non-treated cells (Control), fluconazole-treated (8 µg/mL), or EDTA-treated (1 mM) cells were included as controls. Images taken after 48 h (stressors) and 24 h (urease) incubation at 30 °C and 37 °C. Experiment performed in biological triplicate and technical duplicate.

To identify mechanisms that may explain the observed inhibitory effects of different extracts, *C. neoforman*s cells were briefly treated with sub-MIC concentrations of extracts and exposed to osmotic, membrane and oxidative stressors. This evaluation revealed that protein extracts from *C. nemoralis*, *P. pilsbryi* and clarified extracts from *C. chinensis* affect cryptococcal ability to resist osmotic stress produced by an excess of NaCl (1.5 M) (Fig. 6a). However, when exposed to H_2_O_2_ (3 mM), a generator of reactive oxygen species (ROS), only clarified extracts from *P. pilsbryi* impaired the survival of cryptococcal cells (Fig. 6b). Lastly, only clarified extracts from *C. chinensis* showed a considerable disturbance effect on membrane stability in the presence of the chaotropic agent, SDS (0.01%) (Fig. 6c). These effects were comparable to 8 µg/mL (1 MIC) of fluconazole, a known membrane disruptor. In all cases, impact of mollusk extracts was affected by the temperature, being more notable at 37 °C. Moreover, to assess potential disruptions in the *C. neoformans* secretory pathway upon extract treatment, we evaluated the production of urease across the treatments at 30 °C and 37 °C (EDTA was used as a control) (Fig. 6d). We did not observe a noticeable reduction in urease production, indicating that extract treatment does not influence the associated secretory pathway of *C. neoformans*.

### Proteomic profiling reveals mollusk-specific proteome signatures supporting phenotypic modulation by extracts

**Figure 7:**
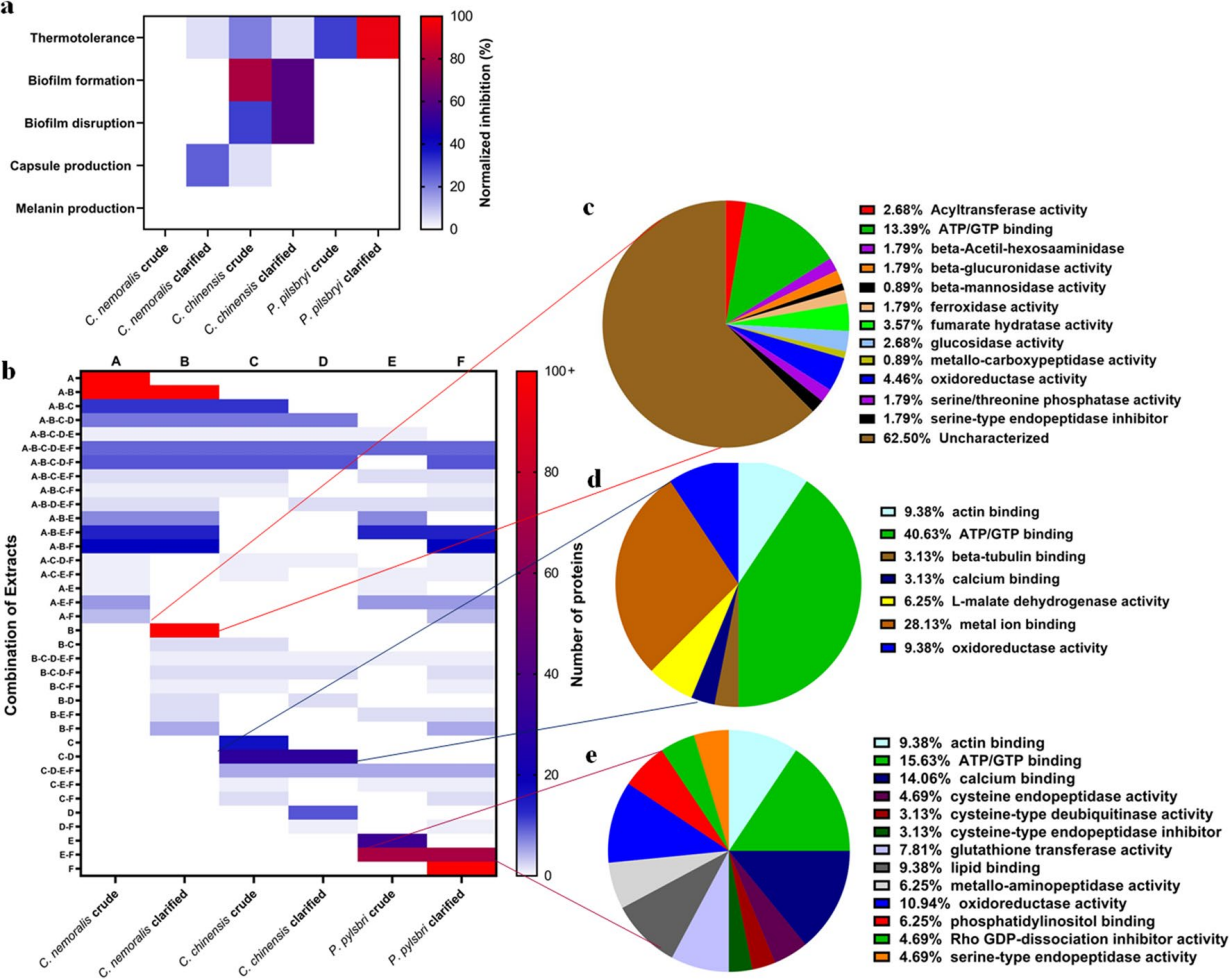
Correlation of phenotypic effects towards *C. neoformans* with proteome signature analysis of mollusk extracts. (**a**) Inhibitory effect of protein extracts on *C. neoformans* virulence factors. Values were normalized to the respective control. (**b**) Number of proteins shared or unique among mollusk extract combinations. (**c**), (**d**), and (**e**) Pie charts show distribution of proteins identified in the mollusk extracts, i.e., *C. nemoralis, C. chinensis* and *P. pilsbryi*, respectively, based on Gene Ontology Molecular Function (GOMF). Proteomics data analyzed with MaxQuant and Perseus. Experiment performed in biological quadruplicate. Figures were prepared using GraphPad Prism 9 (https://www.graphpad.com/scientific-software/prism/).

To identify potential candidate proteins present in the mollusk extracts, bottom-up mass spectrometry-based proteomic profiling was performed. This analysis revealed more than 1200 proteins across the three mollusk species extracts (crude and clarified). To begin, we normalized the specific mollusk effects towards *C. neoformans* virulence factor production based on our phenotypic observations outlined above and summarized the findings (Fig. 7a). For thermotolerance, all mollusk extracts, except crude *C. nemoralis*, had an impact on fungal growth with the clarified *P. pilsbryi* showing the highest impact. Biofilm disruption and formation was impacted by *C. chinensis* (crude and clarified) extracts but unaltered in the presence of the other extracts. Capsule production was influenced by clarified *C. nemoralis* and to a lower extent, both extracts of *C. chinensis.* None of the extracts altered melanin production.

Next, we defined each of the mollusk extracts (i.e., *C. nemoralis, C. chinensis, P. pilsbryi*) and the respective preparation (i.e., crude, clarified) from A-F (Supplementary Table S2) and identified proteome signatures for each combination (Fig. 7b). Unique and common protein identification numbers for each classification ranged from 0 to more than 100. To explore the potential relationship between proteome profiles and phenotypic changes to *C. neoformans* virulence factor production, we used Gene Ontology Molecular Function (GOMF) affiliations. For instance, clarified extracts from *C. nemoralis* (role in inhibition of capsule production) includes 406 unique proteins, such as ATP binding (approx. 13.4%), β-hexosaminidases (approx. 5%), and serine peptidase inhibitors (approx. 1.79%) (Fig. 7c). Similarly, extracts from *C. chinensis* (role in inhibition of fungal biofilms) share 30 proteins in common between the crude and clarified extracts with roles in ATP binding (approx. 40.6%), metal binding (approx. 28.1%), and cytoskeleton interaction (approx. 9.4%) (Fig. 7d). Likewise, extracts from crude and clarified *P. pilsbryi*, with effects on fungal growth and thermotolerance, share 71 proteins involved in multiple biological processes, such as lipid binding (approx. 9.4%), actin binding (approx. 9.4%), and cysteine-like peptidase inhibitors, (approx. 3.1%) (Fig. 7e). Together, we observed GOMF-associated proteome signatures for each extract connected with fungal virulence factor production, supporting a foundation for further exploration into specific proteins driving these observations to directly correlate mollusk protein production and fungal anti-virulence.

## Discussion

Without an adaptive immune system, invertebrates (e.g., mollusks) have developed an extensive array of chemical defenses against environmental pathogens, which are regulated to prevent disruption to the host^21,45^. These properties highlight invertebrates as potential sources of new compounds with specific antimicrobial activity against human fungal pathogens (e.g., *C. neoformans*), which share target similarity with humans^46^. During the last 20 years, multiple compounds, including antimicrobial peptides and peptidase inhibitors, have been identified with activity against human fungal pathogens^9^. However, most investigations focused on killing the pathogen (i.e., fungicidal effect) rather than inhibiting virulence factors (i.e., fungistatic effect), and only a few have been reported from invertebrates^47^. These fungicidal compounds exert high selective pressure and promote the development of resistance mechanisms, affecting long-term clinical uses. Therefore, investigation into alternative strategies (i.e., anti-virulence) that include disarming the pathogen, thus reducing selective pressure towards resistance, presents an attractive (and underexplored) avenue for new antifungal discovery^48^.

### Effects on growth and thermotolerance

*C. neoformans* is a mesophilic fungus, ubiquitous across environments, such as eucalyptus trees and pigeon droppings^49^. Although optimal fungal growth temperature is defined at 30 °C, the pathogen has adapted to the human physiological temperature of 37 °C through elaboration of diverse virulence factors^6^. Here, we observed that crude extracts from *C. chinensis* delayed the growth of cryptococcal cells, especially at 37 °C; however, the effect was diminished after clarification, indicating a loss of influential proteins of high molecular weight. Further analysis revealed that crude extracts from *C. chinensis* do not influence membrane or oxidative stress, indicating another molecular mechanism driving the observations. In this context, like other yeasts*, C. neoformans* uses an α-factor crucial for mating and reproduction^50^, which is synthesized as a pro-α-factor that requires a proteolytic activation by Kex-2 (belonging to the S8B subtilisin family)^51,52^. From the mollusk extracts, we identified subtilisin-like inhibitors with IC_50_ values eight times lower than the *C. chinensis* concentrations used, supporting our findings of reduced fungal growth in the presence of this mollusk extract.

For *P. pilsbryi*, extracts showed a significant reduction in cryptococcal cell growth. In this case, clarified extracts exhibited higher growth inhibition compared to crude extracts, indicating a successful loss of contaminants after thermal precipitation. Further, the fungicidal effect showcased an apparent dose-dependent behavior, suggesting the presence of growth inhibitors in both extracts^53^. While crude extracts did not inhibit growth at 30 °C, this effect appeared at 37 °C; a similar result obtained with *C. chinensis* extracts. The absence of membrane effects with a notable impact on osmotic stress suggests that growth inhibition by *P. pilsbryi* extracts is associated with membrane pore formation, especially at 37 °C. Interestingly, our detection of proteins (approx. 9.4%) with lipid binding activity in *P. pilsbryi* extracts supports such a mechanism and may explain inhibitory effects on growth. Similarly, clarified *P. pilsbryi* extracts may have fungicidal effects against *C. neoformans* driven by impairing antioxidative mechanisms and promoting susceptibility to oxidative stress.

These data suggest either the presence of compounds that impair *C. neoformans* thermotolerance or, a protective effect against the active molecules in the extracts is repressed to help the fungus cope with the higher temperature. For instance, to survive at physiological temperatures, *C. neoformans* produces multiple heat-shock proteins and transcription factors^54^. This process requires the acquisition of nutrients, especially amino acids, from the extracellular space, highlighting a role for proteolytic enzymes to interfere with these nutrients. In this context, the fungus uses multiple enzymes, such as a serine Carboxypeptidase D (CNAG_00919) to digest extracellular and host proteins^55,56^. Using a combination of mass spectrometry-based proteomics approach and biochemical assays, we detected the presence of serine peptidase inhibitors (IC_50_ 1.9 µg/mL) that may be involved in fungal growth inhibition. Altogether, these results suggest the presence of Kunitz-type proteins, which are known for dual activity against serine peptidases and ion channel interaction^57^. Conversely, yeast cells may undergo autolysis, providing nutrients to other cells^58^, which may explain why a diminished OD_600_ at 37 °C after 24 h was observed with clarified *P. pilsbryi* extracts. While these IC_50_ values cannot be compared quantitively^58^, similar results have been observed for peptidase inhibitors from plants with antimicrobial and antifungal activity^59,60^.

### Effects on biofilm formation and disruption

Biofilms are highly dense structures created by microorganisms as a protective mechanism against the host immune system, environmental stress, and antimicrobial molecules (e.g., drugs)^61^. Cryptococcal biofilms are a current threat due to their common appearance in clinical settings and difficulty to treat^62^. In this study, we found that protein extracts from *C. chinensis* impaired the formation of cryptococcal biofilms and we also observed a significant impact (p < 0.05) on capsule production by these extracts, which can affect polysaccharide release, adherence and thus, biofilm formation^63^. A similar result was observed using the antifungal, Amphotericin B, which relies on this inhibitory mechanism to impair cryptococcal biofilms^64^. Likewise, stress assays suggest that clarified extracts from *C. chinensis* disrupt biofilms by membrane perturbation mechanisms, such as pore formation, membrane instability and cell lysis. Furthermore, *C. neoformans* uses an extracellular A1 aspartic peptidase (CNAG_05872) involved in metabolic activity and stress response modulation for biofilm formation^62^. Notably, we detected a moderate inhibitory effect on pepsin-like peptidases in clarified extracts of *C. chinensis* (IC_50_ 15.55 µg/mL), but not in the crude extract, which is consistent with the phenotypic effects observed. These findings suggest the presence of different active molecules and inhibitory mechanisms against cryptococcal biofilm between crude and clarified extracts.

Intriguingly, protein extracts from *C. chinensis* also showed extensive disruption on pre-formed cryptococcal biofilms at 37 °C. In this context, Pqp1 (CNAG_00150), a membrane-associated S8 subtilisin-like peptidase, is involved in quorum-sensing mechanisms in highly compact structures and production of other proteolytic enzymes directly related with virulence in *C. neoformans*^9,65^. Likewise, we determined strong inhibitory effects against subtilisin-like peptidases (IC_50_ around 5 µg/mL) in both extracts from *C. chinensis*, which may explain our findings against biofilm disruption. Overall, these results highlight the potential of active compounds in the clarified extract against this important virulence factor with impacts for clearance of pre-established biofilms from within the host or on medical devices.

### Effects on capsule production

Cryptococcal cells produce a polysaccharide capsule composed mainly of glucuronoxylomannan (GXM) and galactoxylomannan (GalXM), which are crucial for fungal resistance against macrophage phagocytic action^66^. We found that only clarified extracts from *C. nemoralis* and both crude and clarified extracts from *C. chinensis* reduced capsule size. Capsule production in *C. neoformans* depends on multiple factors, such as the Rim101 complex, part of the RIM pathway involved in pH sensing^48^. This transcriptional regulator requires the proteolytic activation of a cysteine peptidase, Rim13 (CNAG_05601). Interestingly, a major difference between crude and clarified extracts of *C. nemoralis* is the unique presence of a moderate cysteine (papain)-like peptidase inhibitor (IC_50_ 57.2 µg/mL) in the clarified extract that support these findings. Moreover, analysis of secretory pathway impacts suggest that extract inhibitory effects may be associated with capsule degradation or impairment of capsule attachment rather that affecting GXM synthesis and/or secretion mechanisms. A lack of impact towards *C. neoformans* secretory pathway was also confirmed by uninterrupted urease secretion observed across the treatments. In this context, around 5% of proteins found in the *C. nemoralis* extracts are chitinase-like enzymes (β-hexosaminidases), which could be involved in the degradation of the capsule as chitinases are naturally produced by invertebrates to digest fungi and/or microorganisms to defend themselves against pathogens like *C. neoformans*^29^.

### Effect on melanin production

Melanin is a dark pigment produced by *C. neoformans* as a defense mechanism against oxidative stress^68^ and antifungal drugs^69^, and to harvest thermal energy within the host^70^. Despite conducting multiple melanin assays, none of the extracts showed a significant (p > 0.05) inhibition of melanin production. Given that melaninization is a normal process during shell formation in mollusks, it is possible that these organisms have not developed a protective mechanism against melanin production based on potentially harmful effects to the mollusk itself^71^. Although, we did observe inhibitory activity towards melanin-associated enzymes (Kexin-like) for crude *C. nemoralis* and *P. pilsbryi*, this activity was lost during clarification and did not impact melanin production by *C. neoformans* as evaluated in this study, suggesting instability of the inhibitors or alternative modes of protection.

### Proteomics profiling of mollusk extracts

Mass spectrometry-based proteomics provides a robust and reliable platform for identifying and quantifying proteins within diverse biological samples^72^. For infectious diseases and profiling of fungal pathogens, applications of proteomics are underutilized but show substantial promise for the discovery of novel drug targets and/or characterization of new virulence factors^24,73,74^. In our study, we focused on opportunities to explain unique phenotypic differences for the mollusk extracts while acknowledging the limitations that the entire proteome of each mollusk was not amendable to profiling (e.g., proteins not produced, not detected in the mass spectrometer, not conducive to trypsin digestion) or database completeness (e.g., genus vs. species alignment for available genomes). Although these limitations restrict the comprehensive picture for each extract, our approach allows us to identify categorical differences and observe abundance changes in protein production across the extracts. With this information, we can propose connections between the observed anti-virulence traits of the extracts from *C. nemoralis*, *P. pilsbryi*, and *C. chinensis*, in relation to crude or clarified preparations, and define protein signatures that may be driving these traits.

## Conclusions

Mollusks are highly diverse sources of natural compounds with promising antifungal activities. In this work, we performed phenotypic characterization of multiple virulence factors of the human fungal pathogen, *C. neoformans*, in the presence of protein extracts from three snail species. Clarified extracts of *P. pilsbryi* have a fungicidal effect on cryptococcal cells, as they may produce membrane pores and osmotic deregulation, as well as nutrition and mating impairment. Similarly, protein extracts of *C. chinensis* can affect cryptococcal thermotolerance, as well as inhibit and disrupt biofilm formation and reduce capsule production, potentially through membrane disruption and inhibition by proteolytic enzymes involved in virulence factor regulation. Lastly, we detected the presence of five chitinase-like enzymes and cysteine peptidase inhibitors that may explain the inhibitory effect of clarified extracts from *C. nemoralis* on capsule production. Together, these results highlight the potential of invertebrates as promising sources for compounds with strong anticryptococcal activity, through consideration of the relationship among the environment, animal, and human health under a One Health lens. Likewise, this study stresses the importance of further steps to purify active molecules from each extract and assess specific antifungal properties specifically targeted to fungal virulence factors instead of direct killing of the pathogen to lower the selective pressure towards resistance.

## Supplementary Information

Supplementary Figure S1.

Supplementary Table S1.

Supplementary Table S2.

Supplementary Table S3.

## Data Availability

The. RAW and affiliated files were deposited into the publicly available PRIDE partner database for the ProteomeXchange^75^ consortium with the data set identifier: PXD038987 https://www.ebi.ac.uk/pride/archive/projects/PXD038987.
