# Supplementary material for: Differentiated extracts from freshwater and terrestrial mollusks inhibit virulence factor production in Cryptococcus neoformans
Source: Sci Rep. 2023 Mar 26;13:4928. doi: 10.1038/s41598-023-32140-3 (PMC10040410; doi:10.1038/s41598-023-32140-3)
Supplement: Supplementary file 1 — Supplementary Figure S1. [file 41598_2023_32140_MOESM1_ESM.docx]

**
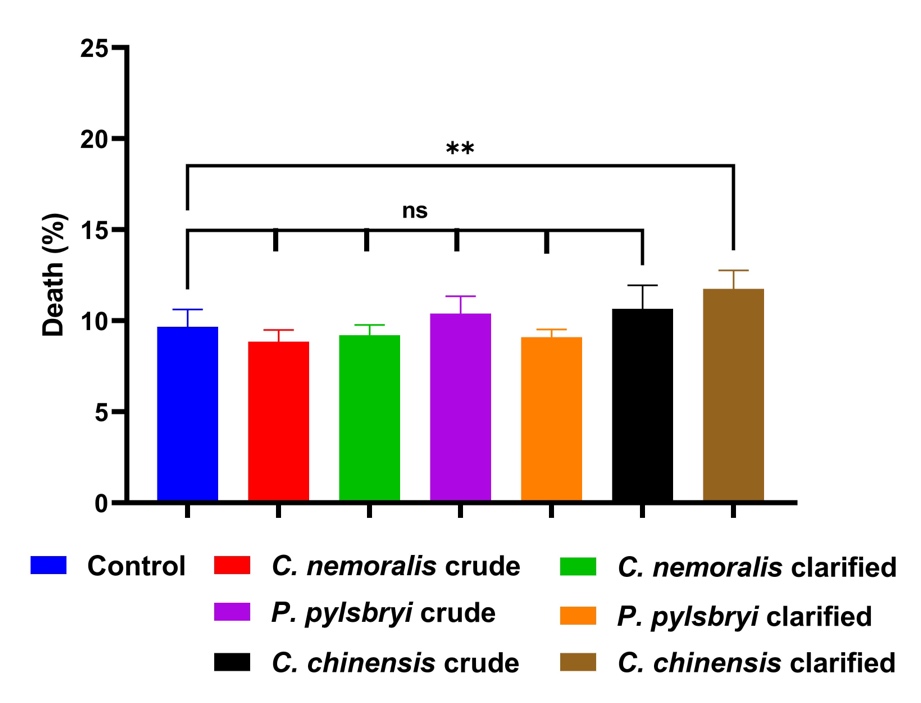
**

**Figure S1:** **Cytotoxicity of protein extracts against BALB/c macrophages.** Bars show percentage (%) of killed macrophages after 4 h incubation with protein extracts from *C. nemoralis* crude (21 µg/mL), *C. nemoralis* clarified (9 µg/mL), *C. chinensis* crude (153 µg/mL), *C. chinensis* clarified (26 µg/mL), *P. pilsbryi* crude (3 µg/mL) and *P. pilsbryi* clarified (3 µg/mL). Control indicates natural death after incubation period. Error bars indicate standard deviation. Statistical analysis was performed using a one-way ANOVA and a Dunnett’s multiple comparison test with a p-value of 0.05. **: p<0.01. Experiment was performed using 6 biological and two technical replicates. Figures were created using GraphPad Prism 9.
