# Supplementary material for: Differentiated extracts from freshwater and terrestrial mollusks inhibit virulence factor production in Cryptococcus neoformans
Source: Sci Rep. 2023 Mar 26;13:4928. doi: 10.1038/s41598-023-32140-3 (PMC10040410; doi:10.1038/s41598-023-32140-3)
Supplement: Supplementary file 2 — Supplementary Table S1. [file 41598_2023_32140_MOESM2_ESM.docx]

| **Enzyme** | **Substrate** | **Km**  **(mM)** | **Buffer and Temperature** | **Excitation/ Emission** | **Reference** |
| --- | --- | --- | --- | --- | --- |
| Carboxypeptidase D  (Creative Enzyme) | furylacryloyl-AK (Sigma) | 0,962 | 50 mM acetate buffer, pH 4.5, 1 mM EDTA, 25 ˚C | 340 (chromogenic) | ^1^ |
| Kexin  (Creative Enzyme) | Boc-QGR-MCA (BioSource) | 0,32 | 50mM Tris-HCl pH 7.0, 1 mM CaCl_2_ 37 ˚C | 328/393 | ^2^ |
| Pepsin from porcine (Sigma) | MOCAc-APAKFFRLK-(Dnp)-NH (BioSource) | 6,17x10^-3^ | 50 mM sodium citrate pH:3 37 ˚C | 328/393 | ^3^ |
| Subtilisin A  (Sigma) | Suc-AAPF-pNA (Sigma) | 0,2 | Activity: 100 mM Tris-HCl pH:8.6, 25 ˚C | 405 nm (chromogenic) | ^4^ |
| Papain  (Sigma) | Z-FR-AMC  (MD Systems) | 0,06 | 100 mM sodium phosphate buffer, pH 6.5, 2 mM DTT, 1 mM EDTA, 25 ˚C | 353/442 | ^5^ |
| Thermolysin (Promega) | Mca-PLGL-Dpa-AR-NH2  (Boc Science) | 2 x 10^-3^ | 50mM Tris-HCl pH 7.5, 1 mM ZnCl_2_ T: 25 ˚C | 328/393 | ^6^ |
| 20S Proteasome from Rat (Fisher Scientific) | Suc-LLVY-AMC (Fisher Scientific) | 0.07 | 50 mM Tris–HCl and 1 mM EDTA, pH 7.5 T: 37 ˚C | 353/442 | ^7^ |

**References:**

1. Latchinian-Sadek, L. & Thomas, D. Y. Expression, purification, and characterization of the yeast KEX1 gene product, a polypeptide precursor processing carboxypeptidase. *J. Biol. Chem.* **268**, 534–540 (1993).

2. Rockwell, N. C. & Fuller, R. S. Interplay between S1 and S4 subsites in Kex2 protease: Kex2 exhibits dual specificity for the P4 side chain. *Biochemistry* **37**, 3386–3391 (1998).

3. Kondo, H. *et al.* Substrate specificities and kinetic properties of proteinase A from the yeast Saccharomyces cerevisiae and the development of a novel substrate. *J. Biochem.* **124**, 141–147 (1998).

4. Wells, J. A., Cunningham, B. C., Graycar, T. P. & Estell, D. A. Recruitment of substrate-specificity properties from one enzyme into a related one by protein engineering. *Proc. Natl. Acad. Sci. U. S. A.* **84**, 5167–5171 (1987).

5. Redzynia, I. *et al.* Crystal structure of the parasite inhibitor chagasin in complex with papain allows identification of structural requirements for broad reactivity and specificity determinants for target proteases. *FEBS J.* **276**, 793–806 (2009).

6. Marguerre, A. K. & Krämer, R. Lanthanide-based fluorogenic peptide substrate for the highly sensitive detection of thermolysin. *Bioorganic Med. Chem. Lett.* **19**, 5757–5759 (2009).

7. Dahlmann, B., Ruppert, T., Kloetzel, P. M. & Kuehn, L. Subtypes of 20S proteasomes from skeletal muscle. *Biochimie* **83**, 295–299 (2001).
