# Supplementary material for: Differentiated extracts from freshwater and terrestrial mollusks inhibit virulence factor production in Cryptococcus neoformans
Source: Sci Rep. 2023 Mar 26;13:4928. doi: 10.1038/s41598-023-32140-3 (PMC10040410; doi:10.1038/s41598-023-32140-3)
Supplement: Supplementary file 3 — Supplementary Table S2. [file 41598_2023_32140_MOESM3_ESM.docx]

**Table S2: Extract combinations used in quantitative proteomics comparisons.**

| A | *C. nemoralis* crude |
| --- | --- |
| A-B | *C. nemoralis* crude-*C. nemoralis* clarified |
| A-B-C | *C. nemoralis* crude-*C. nemoralis* clarified-*C. chinensis* clarified-*C. chinensis* crude |
| A-B-C-D | *C. nemoralis* crude-*C. nemoralis* clarified-*C. chinensis* crude-*C. chinensis* clarified |
| A-B-C-D-E | *C. nemoralis* crude-*C. nemoralis* clarified-*C. chinensis* crude-*C. chinensis* clarified-*P. pilsbryi* crude |
| A-B-C-D-E-F | *C. nemoralis* crude-*C. nemoralis* clarified-*C. chinensis* crude-*C. chinensis* clarified-*P. pilsbryi* crude-*P. pilsbryi* clarified |
| A-B-C-D-F | *C. nemoralis* crude-*C. nemoralis* clarified-*C. chinensis* crude-*C. chinensis* clarified-*P. pilsbryi* clarified |
| A-B-C-E-F | *C. nemoralis* crude-*C. nemoralis* clarified-*C. chinensis* clarified-*C. chinensis* crude-*P. pilsbryi* crude-*P. pilsbryi* clarified |
| A-B-C-F | *C. nemoralis* crude-*C. nemoralis* clarified-*C. chinensis* clarified-*C. chinensis* crude-*P. pilsbryi* clarified |
| A-B-D-E-F | *C. nemoralis* crude-*C. nemoralis* clarified-*C. chinensis* clarified-*P. pilsbryi* crude-*P. pilsbryi* clarified |
| A-B-E | *C. nemoralis* crude-*C. nemoralis* clarified-*P. pilsbryi* crude |
| A-B-E-F | *C. nemoralis* crude-*C. nemoralis* clarified-*P. pilsbryi* crude-*P. pilsbryi* clarified |
| A-B-F | *C. nemoralis* crude-*C. nemoralis* clarified-*P. pilsbryi* clarified |
| A-C-D-F | *C. nemoralis* crude-*C. chinensis* crude-*C. chinensis* clarified-*P. pilsbryi* clarified |
| A-C-E-F | *C. nemoralis* crude-*C. chinensis* crude-*P. pilsbryi* crude-*P. pilsbryi* clarified |
| A-E | *C. nemoralis* crude-*P. pilsbryi* crude |
| A-E-F | *C. nemoralis* crude-*P. pilsbryi* crude-*P. pilsbryi* clarified |
| A-F | *C. nemoralis* crude-*P. pilsbryi* clarified |
| B | *C. nemoralis* clarified |
| B-C | *C. nemoralis* clarified-*C. chinensis* crude |
| B-C-D-E-F | *C. nemoralis* clarified-*C. chinensis* crude-*C. chinensis* clarified-*P. pilsbryi* crude-*P. pilsbryi* clarified |
| B-C-D-F | *C. nemoralis* clarified-*C. chinensis* crude-*C. chinensis* clarified-*P. pilsbryi* clarified |
| B-C-F | *C. nemoralis* clarified-*C. chinensis* crude-*P. pilsbryi* clarified |
| B-D | *C. nemoralis* clarified-*C. chinensis* clarified |
| B-E-F | *C. nemoralis* clarified-*P. pilsbryi* crude-*P. pilsbryi* clarified |
| B-F | *C. nemoralis* clarified-*P. pilsbryi* clarified |
| C | *C. chinensis* crude |
| C-D | *C. chinensis* crude-*C. chinensis* clarified |
| C-D-E-F | *C. chinensis* crude-*C. chinensis* clarified-*P. pilsbryi* crude-*P. pilsbryi* clarified |
| C-E-F | *C. chinensis* crude-P. pylsbri crude-P. pylsbri clarified |
| C-F | *C. chinensis* crude-*P. pilsbryi* clarified |
| D | *C. chinensis* clarified |
| D-F | *C. chinensis* clarified-*P. pilsbryi* clarified |
| E | *P. pilsbryi* crude |
| E-F | *P. pilsbryi* crude-*P. pilsbryi* clarified |
| F | *P. pilsbryi* clarified |
